# Probing the design principles of photosynthetic systems through fluorescence noise measurement

**DOI:** 10.1038/s41598-024-64068-7

**Published:** 2024-06-16

**Authors:** Naama Maroudas-Sklare, Naama Goren, Shira Yochelis, Grzegorz Jung, Nir Keren, Yossi Paltiel

**Affiliations:** 1 Department of Applied Physics, Hebrew University of Jerusalem, 91904 Jerusalem, Israel; 2 Department of Plant & Environmental Sciences, The Alexander Silberman Institute of Life Sciences, Hebrew University of Jerusalem, Jerusalem, Israel; 3 Department of Physics, Ben Gurion University of the Negev, 84105 Beer Sheva, Israel; 4 Instytut Fizyki PAN, 02668 Warszawa, Poland

**Keywords:** Biological fluorescence, Plant sciences, Characterization and analytical techniques

## Abstract

Elucidating the energetic processes which govern photosynthesis, the engine of life on earth, are an essential goal both for fundamental research and for cutting-edge biotechnological applications. Fluorescent signal of photosynthetic markers has long been utilised in this endeavour. In this research we demonstrate the use of fluorescent noise analysis to reveal further layers of intricacy in photosynthetic energy transfer. While noise is a common tool analysing dynamics in physics and engineering, its application in biology has thus far been limited. Here, a distinct behaviour in photosynthetic pigments across various chemical and biological environments is measured. These changes seem to elucidate quantum effects governing the generation of oxidative radicals. Although our method offers insights, it is important to note that the interpretation should be further validated expertly to support as conclusive theory. This innovative method is simple, non-invasive, and immediate, making it a promising tool to uncover further, more complex energetic events in photosynthesis, with potential uses in environmental monitoring, agriculture, and food-tech.

## Introduction

Photosynthetic processes are of paramount global importance^1^. They serve as the primary mechanism for capturing solar energy and transforming it into usable forms, sustaining life on Earth by producing oxygen and providing the foundation for food chains and ecosystems^1,2^. Understanding the underlying principles of photosynthesis is crucial for tackling pressing global challenges such as climate change and food security^3^. Contemporary advancements in research methodologies have greatly enhanced our ability to investigate and characterize photosynthesis^4–12^. In recent years, quantum mechanics has provided new avenues of scientific inquiry^13–20^. An interesting question that results from quantum biological models is the influence of classical and quantum noise on photosynthetic performance^18,19,21^.

The first step of the photosynthetic processes, and the one most prone to manifest quantum effects, is capturing and funneling light energy into the photochemical centers^20^. This function is performed by photosynthetic pigment-protein light-harvesting complexes (LHCs), cyanobacterial phycobilisomes (PBS) and plant LHCs, for example. They are composed of pigment molecules, primarily chlorophylls, phycobilins and carotenoids, organized into intricate protein matrixes^2,22–24^. In cyanobacteria, phycobilins are arranged into massive PBS structures^25^. Chlorophyll (Chl) containing LHCs are integrated into the thylakoid membranes of chloroplasts. Their structure is organized in a way that can be optimized for light absorption and energy transfer, while allowing for flexibility in response to changing environmental conditions^26,27^.

The primary function of light harvesting apparatus is to control energy transfer to photochemical reaction centers. Although absorbed energy is inevitably lost as heat and fluorescence, these losses can be utilized to protect the organism from the oxidative stress associated with fully reduced (closed) photosystems^1,9,27^. Elucidating the structure and function of photosynthetic antenna systems is crucial for unraveling the mechanisms of light capture and energy conversion in photosynthesis^28–30^. Absorbed light excites Chl molecules to excited states in the visible range- the first and second excited singlet states^31^.

**Figure 1:**
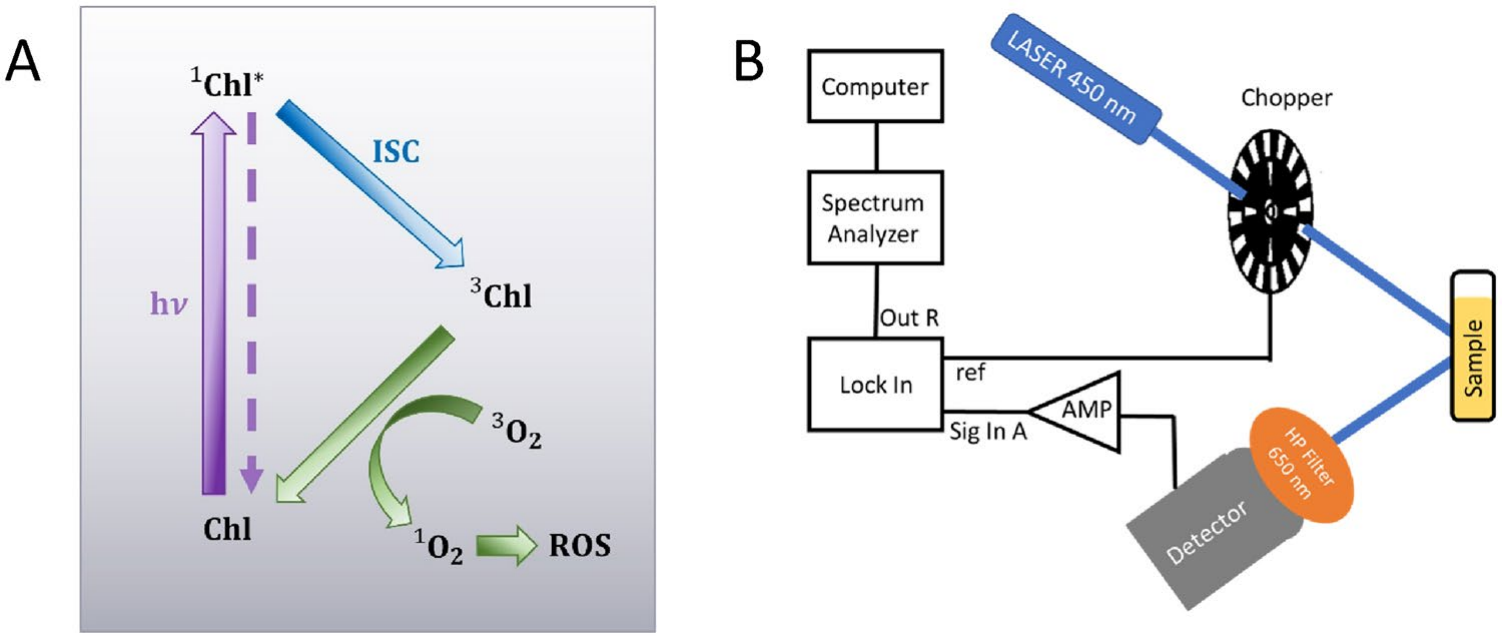
(**A**) Diagram of Chl excited states and the interaction of triplet Chl with triplet oxygen to create singlet oxygen, leading to further creation of harmful ROS. (**B**) Measurement setup.

If there is an excess of singlet states in a photosynthetic system, an over-exited population can be created. Over-excitation increases the probability for longer-lived Chl triplet states $({}_{{}}^{3} {\text{Chl}}^{*}$) by intersystem crossing (ISC)^32,33^. An excess of singlet states in a photosynthetic unit (defined as the subset of pigments contributing energy to a single RC^34^), is characterized by a state in which the rate of exciton production on by light harvesting pigments exceeds the rate of charge separation in the RC. Under physiological conditions these rates do not amount to a level where singlet–singlet annihilation processes play a major role. In that situation, reactions with the ${}_{{}}^{3} {\text{Chl}}^{*}$ may generate intensely harmful molecular species: either free radicals by electron or hydrogen transfer (type I mechanism), or highly reactive singlet oxygen by interaction with oxygen in its triplet ground state (type II mechanism)^35–37^ (Fig. 1A). These reactive oxygen species (ROS) can irreversibly damage the organism^1^. Throughout evolution, photosynthetic organisms developed multiple coping mechanisms to avoid damage^38–40^. Carotenoids, which are components of many LHCs, provide essential photoprotection by efficiently quenching ${}_{{}}^{3} {\text{Chl}}^{*}$ through energy transfer enabled by the Van der Waals (VdW) proximity of the molecules^12,32,33,41–43^.

It is well known that the lifetimes of singlet and triplet Chl, and the rates of ISC which enable ${}_{{}}^{3} {\text{Chl}}^{*}$ formation in the first place, are dependent on the solvent^31,44–47^. It therefore follows that the diverse protein and membrane environments found in biological organisms would also influence these lifetimes and ISC rates^44,48–50^. Fine-tuning of the molecular environment within photosynthetic organisms could be utilized to limit ${}_{{}}^{3} {\text{Chl}}^{*}$. The mechanisms behind this process are still under investigation^47^.

Fluorescence measurements serve as powerful tools for assessing photosynthetic performance^7,51,52^. These measurements primarily focus on capturing the intensity or the lifetime of the signal^53^, but measurements of the associated noise can yield yet more valuable information. Noise provides a robust framework for characterizing and modelling stochastic processes. This comprehensive approach allows for a deeper understanding of complex systems and offers insights into various processes governed by randomness, including structural changes^54^, preferential growth, unbalanced reactions, and the occurrence of rare events^54,55^.

A fundamental characterization of the statistical properties of the noise is the determination of its Gaussian or non-Gaussian nature. Gaussian noise is characterized by a symmetric probability distribution arising from the central limit theorem and can arise from environmental variables and a multitude of independent and identically distributed sources^56^, such as thermal fluctuations or measurement errors. On the contrary, non-Gaussian noise exhibits asymmetric distributions, suggesting the presence of intricate underlying dynamics and interactions^57^. Non-Gaussian noise can emerge from a variety of sources, including the combination of separate Gaussian components^58^, types of random walks^59^, or avalanche-like phenomena^60^. Furthermore, it can originate from nonlinear parameters and quantum effects^61^.

Quantum noise, rooted in the inherent uncertainty associated with quantum mechanics, manifests with frequency asymmetry and zero-point motion^58^. Signals from several quantum levels that are separated enough compared to thermal energy are also expected to be non-Gaussian.

In biological systems, noise can arise from both classical and quantum sources, particularly in processes occurring at the nanoscale^21,54,62^. Classical noise encompasses contributions from environmental factors, molecular interactions, or fluctuations in cellular components. These noise sources can exhibit Gaussian or non-Gaussian distributions, depending on the underlying processes^63^. On the other hand, quantum noise arises from quantum mechanical effects such as the discrete nature of energy levels^64^, spin states, tunnelling^65^, many-body dynamics^66–71^, or quantum coherence^72^. Noise has also been used to detect excitation energy transfer^73,74^. By considering both classical and quantum sources of noise, a more comprehensive understanding of the complex dynamics of biological systems can be attained.

Here, we perform noise analysis of the fluorescence signal from photosynthetic pigments both in vitro and in vivo, to gain a more comprehensive understanding of underlying mechanisms of singlet to triplet transitions in light harvesting complexes. The time scale of excitation energy transfer in photosynthesis is on the order of femtoseconds (fs) to picoseconds (ps)^75,76^. Detection of these processes is challenging and can be done by electron paramagnetic resonance (EPR) or by ultrafast nonlinear spectroscopy^77–80^, but are hard to achieve in vivo. Here we apply low frequency noise measurements as a simple method to probe much faster dynamics.

Chl pigments are measured in different solvents and oxygenic conditions and compared with those in various organisms in vivo. The dynamics of singlet and triplet Chl (Fig. 1A) are probed by measuring noise, suggesting a mechanism whereby triplet formation is inherently suppressed in photosynthetic systems in vivo.

### Methods

#### Sample preparation

Chl pigments were extracted from *Synechocystis* sp. PCC 6803. Although carotenoids are also extracted with the chlorophyll, they do not fluoresce, hence do not contribute to the signal. The samples were prepared in 80% acetone. PEG (polyethylene glycol 6000 g/mol MW, 40 g/100 ml conc.) samples were prepared from a 1:5 dilution of the acetone extract (labelled Chl-PEG), while acetone (Chl-Acetone) samples were diluted 1:10 with 80% acetone from the original extract. These optically thin dilutions gave the same order of magnitude of final fluorescent signal in our setup, comparable with the magnitude of the in vivo samples. Oxygen depleted samples (labelled as Chl-Ace-Nit) are the 1:10 acetone samples bubbled with nitrogen for 30 min. The in vivo samples include the cyanobacteria *Synechococcus* sp. WH 8102 (Syn8102), *Synechocystis* sp. PCC 6803 (Syn6803), and leaves from *Arabidopsis thaliana* plants (LeafArb). The chlorophyll from cyanobacteria Synechococcus sp. WH8102 naturally contains only chlorophyll a. Fluorescence was measured using a 650 nm high-pass filter (650 HP) to isolate the chlorophyll fluorescence signal (emission peak: 685 nm^81^) as much as possible from those of other pigments present in the in vivo samples (namely phycocyanin in cyanobacteria, which has a emission peak at 650 nm^82^). Fluorescence measurement of Chl-Acetone sample can be found in Fig. S4 in the SI.

#### Measurement setup

The fluorescence and its fluctuations were measured using the setup depicted in Fig. 1B. A laser emitting light at a wavelength of 450 nm served as the light source. An electric chopper was employed, with a modulation frequency set at 111 Hz, well above the upper limit of the noise measuring bandwidth. The sample was held in a quartz cuvette, and the light emitted from the sample was collected through a 650 nm high-pass filter. A detector, positioned perpendicular to the light source, captured the collected light. The detector signal was then amplified with a gain of 10^6 V/A and fed into a lock-in amplifier, with the chopper signal serving as the reference. The output signal of the lock–in was delivered to the computer assisted spectrum analyser. To achieve better resolution at low frequencies, measurements were acquired for approximately 300 s and were repeated four times. The time scale of the experiment is set by the frequency range of the spectra measured that is sub 50 Hz. However, even if the processes in question are out of this range it does not mean that they do not influence the overall noise. The experiments probe the overall changes in spectra.

#### Noise analysis

**Figure 2:**
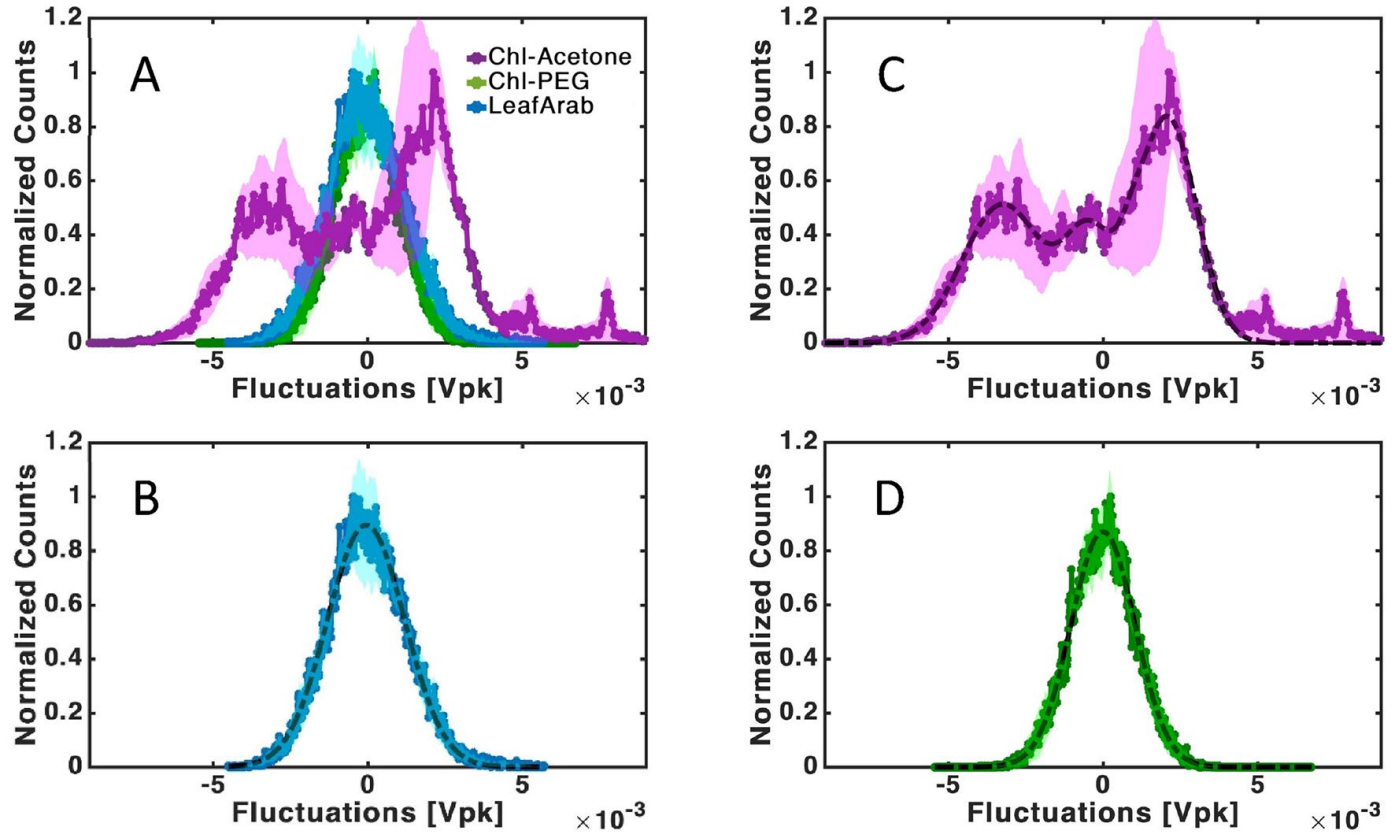
Noise amplitude distribution and fitting: (**A**) Histograms comparing in-vivo LeafArab (blue), in-vitro chlorophyll in an acetone solution (purple), and in-vitro chlorophyll in a PEG solution (green). (**B**–**D**) display each histogram individually with a Gaussian fit (dashed black line), of the first order for (**B**) and (**D**), and of the fourth order for (**C**). Fit coefficients can be found in the Supporting Information (Table 1). Shaded area denotes averaging error. Each histogram is an average of about 10 measurements. The shade area, showing the measurement error is large for Chl-Acetole reflecting the shift in peaks with every measurement.

**Figure 3:**
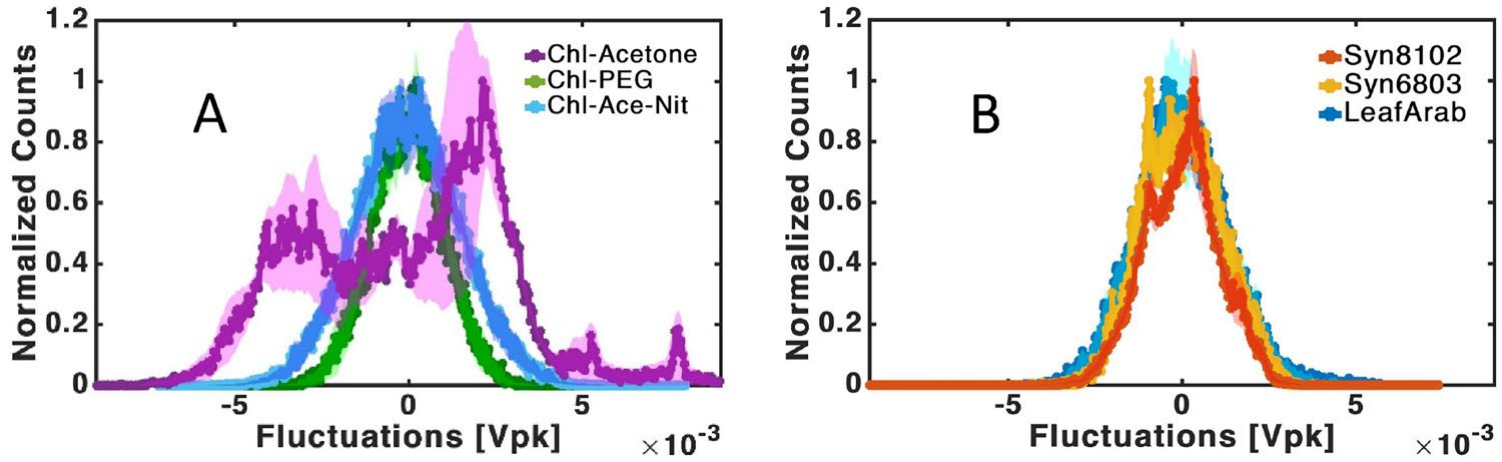
In-vitro Vs in-vivo histograms: (**A**) in-vitro compounds: chlorophyll in an acetone solution—purple, chlorophyll in a PEG solution—green and chlorophyll in an acetone solution after Nitrogen bubbling—light blue. (**B**) in-vivo compounds: Syn8102—orange, Syn6803—yellow and Arab leaf—blue. Shaded area denotes averaging error. Each histogram is an average of about 10 measurements. The shaded area showing the measurement error is large for Chl-Acetone, reflecting the shift in peaks with every measurement.

The noise signal was analysed in time and frequency domain. In the time domains, we have recorded the noise waveforms and acquired histograms of the noise amplitude distribution, to provide insights into the underlying dynamics and sources of variability. Background noise was measured without any sample but with all other setup elements including the chopper, light source, and amplifier. These background noise spectra are presented in the Supporting Information (Figs. S1 and S3). This noise was subtracted from the signal noise. The histograms were calculated after reducing a linear fit, to avoid signal drift effects. The time domain amplitude histogram allows for the initial identification of the statistical properties of the noise. Every histogram presented in Figs. 2 and 3 represents an average of multiple measurements, generating an averaging error, and has been normalized based on its maximum value.

Noise analysis in the frequency domain consisted in power spectral density estimation. This analysis, with an emphasis on the characteristics in the low frequencies, enables the identification of specific phenomena or processes driving the observed fluctuations. The power spectral density estimation provided a quantitative representation range, facilitating further investigation and interpretation of the noise characteristics.

## Results

Here, we perform noise analysis of the fluorescence signal from photosynthetic pigments both in vitro and in vivo in different confining matrices. The most striking differences between the samples are the width and shape of the noise amplitude distributions. Chl-Acetone samples have the widest noise amplitude distribution, followed by Chl-Ace-Nit, while Chl-PEG and all three in vivo samples have similar, narrower distributions (Figs. 2, 3). Most biological processes generate Gaussian distributions, where the normalized width indicates the noise intensity. Where multiple processes are involved, we expect to see multiple peaks of superimposed Gaussian distributions. Surprisingly, the shape of the Chl-Acetone histogram presents a multi-peak Gaussian distribution (Fig. 2C), while all the other samples have a dominant single peak (Fig. 2B,D and Fig. S1 in the SI). Gaussian distribution of noise amplitudes is the most encountered distribution in nature. This simple fit for multiple Gaussian distribution demonstrates the complexity of the system. Multiple Gaussian distributions often indicate the non-Gaussian character of the noise. This is true, for example, for a quantum two level system where each level noise is characterized with a different Gaussian distribution. The multiple Gaussian distribution fits the experimental data very well for the Chl-Acetone sample (Figs. 2C and S2). This is a critical point which correlates the measurement to multiple processes.

## Discussion and suggested mechanism

These results present two issues that need to be explained: (a) the wider distribution with (b) multiple peaks, in Chl-Acetone, as compared to all other samples. These results are suggestive of a multi-level quantum system. As mentioned above, Chl can populate both singlet and triplet excited states (see Fig. 1A). The Chl in acetone seems to be able to access a wider range of excited states, leading to more than one excitation peak and an overall wider distribution. In acetone, Chl has access to higher degrees of freedom (rotation, translation, and vibration) than when in a more viscous solution such as PEG, or when embedded in LHCs and thylakoid membranes in vivo.

The flexibility of motion widens the range of excited states accessed by Chl in acetone, while Chl in PEG or in vivo are limited to one excited state. This dominant state is most probably singlet. Hence, it is possible that photosynthetic evolution selected protein matrix designs that limit movement and curb the formation of its triplet states, thus minimizing the generation of destructive ROS. If this is the case, the free Chl is dominated by multilevel quantum fluctuations, while confined Chl noise can be well fitted to a single-peak Gaussian of the noise distributed around a specific quantum level.

To test this theory, we performed a similar analysis of samples that were bubbled with ${\text{N}}_{2}$ prior to measurement in order to deplete ${\text{O}}_{2}$ within the solution (labelled Chl-Ace-Nit). These samples, as seen in Fig. 3, also show a narrow distribution, although wider compared to that of Chl-PEG and the in vivo samples. A possible explanation for this trend is that although the ${}_{{}}^{3} {\text{Chl}}^{*}$ state is available for the Chl-Ace-Nit samples, the interaction with oxygen, which should also appear as a peak in the histogram, is now inaccessible.

In Chl-Ace-Nit samples, we suggest that the noise amplitude distribution can be explained as a steady state with a fixed number of triplet and singlet states. This combination could appear as the sum of two Gaussians. Meanwhile, in the Chl-PEG and in vivo samples, a depletion of the triplet state and corresponding peak is observed, thus a single Gaussian emerges. Beyond higher viscosity which restricts movement, it is possible that the interaction of Chl with oxygen is further hindered by a lower solubility of oxygen in PEG^83^. These interpretations point to the idea that the full range of possibilities- singlet and triplet Chl, along with their possible interactions with oxygen, is measured in the Chl-Acetone samples, leading to a multi-peak histogram that is composed of different Gaussians relevant to specific processes. It’s important to note that the proposed scenarios represent potential explanations based on the observed data, and do not constitute strict evidence. Additional research is needed to validate these hypotheses.

**Figure 4:**
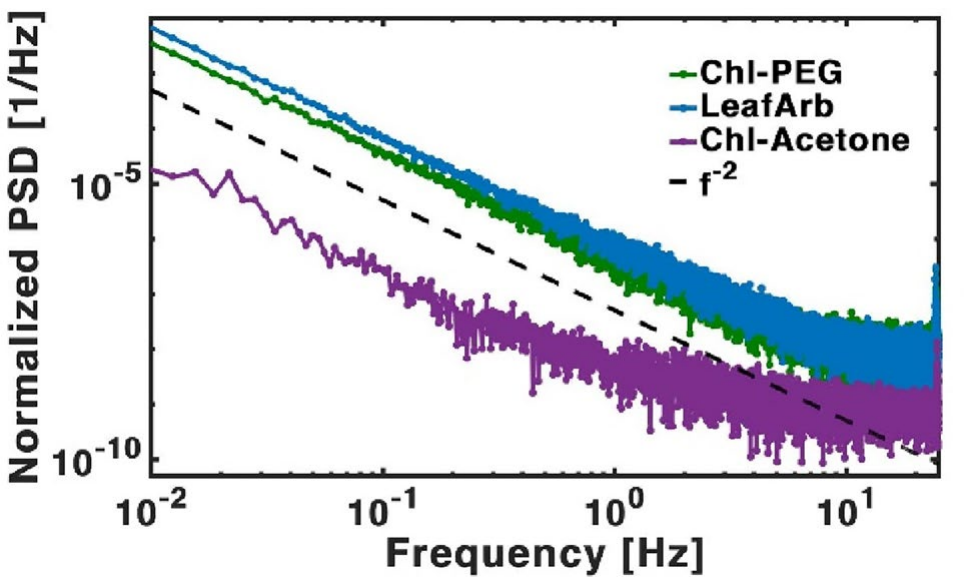
Power spectral density of in-vitro: chlorophyll in an acetone solution—purple, chlorophyll in a PEG solution—green and in-vivo, LeafArb—blue. Dashed black line present a first order power law fit of $f^{ - 2}$.

Further analysis was conducted examining the Power Spectral Density (PSD). Figures 4 and S3 in the SI present an average of PSDs obtained from several time waveforms that were obtained. As shown in Fig. 4, the slope of the spectra was analysed by fitting it to a power-law function of the form $a \cdot f^{b}$. The spectra were normalized by the square of the DC luminescence value of the given sample. Notably, the spectra consist of two contributions: at low frequencies, a $b = - 2$ spectrum is dominant, while at higher frequencies the white noise corresponding to thermal fluctuations is observed, with a typical slope of $b = 0$. The obtained slope at low frequencies was found to be close to − 2 in all samples. A slope of − 2 in the PSD is typical for random walk noise^84^, which signifies a cumulative effect of random fluctuations over time^59,85^. This is a dominating type of noise in biological systems, and has been observed in various contexts including animal paths^86,87^, cell signalling and movement^88^. In photosynthesis, electronic excitation energy undergoes a random walk via a hopping mechanism within the antenna complex until it either becomes trapped by a reaction centre (RC) or is dissipated through heat or fluorescence^10,89^.

It is notable that the spectra of LeafArb and Chl-PEG are almost identical, which supports our hypothesis that the mechanism of Chl triplet suppression is comparable in both these systems—physical constraint of movement. The Chl pigments in both systems are relatively static in space, leading to areas with larger or smaller quenching abilities depending on their physical formation.

## Conclusions

We have presented noise analysis of fluorescence signals from photosynthetic pigments both in vitro and in vivo, to gain a more comprehensive understanding of underlying mechanisms of singlet to triplet transitions in light harvesting complexes. Noise measurements in biology may add an innovative approach for probing excited spin states in both chemical and biological samples, both in vivo and in vitro. The pulsed noise measurements provide a simple way to detect an ultrafast process without the need for complex instrumentation.

Through this study, we propose a hypothesis that biology utilizes membrane embedded protein matrixes to avoid the formation of excited triplet chlorophyll states. The biological membrane in our measurements seems to play a critical role in the photosynthetic processes.

Histogram analysis revealed significant differences in the chlorophyll's fluorescence response depending on their environment. Notably, chlorophyll in acetone displayed additional peaks compared to its behaviour in live membranes or oxygen-depleted solutions. This points to a spin excitation and relaxation mechanism.

Another interesting property is the large difference in the spectra and intensity between the Chl in acetone and the other more complex structures. Since the slope of the spectra and the intensity can point to different dominating processes, these results may show that in the membrane, several diffusion processes are restricted. We are now studying this point in a deeper way.

It is conceivable that through evolution, life was able to narrow its operational territory to a single energy level at which the operational efficiency is maximized, and the organism is best protected from harmful radicals. Noise analysis may probe and point to this exact regime. The novelty of our characterization method lies in its simplicity, non-invasiveness, and immediate results. The ability to perform remote excited state analysis is of great significance in both chemical and biological research.

While noise is a common tool analysing dynamics in physics and engineering, it is not common in biology, supplying a non-intrusive approach that allows for repeated measurements and monitoring over time. As such, it may offer the potential for environmental and agronomical monitoring in longitudinal studies and real-time observations of photosynthetic dynamics. These could include early signals of nutrient and water depletion, as will be published in a following manuscript. If this interpretation is true, future applications of this novel approach range from fundamental quantum biology research to monitoring the growth and health of food crops.

## Supplementary information

Additional hysteresis and PSD spectra of all samples separately, including fit parameters for Gaussian fit to all hysteresis. Absorption signal for Chlorophyll in an Acetone solution.

## Supplementary Information

Supplementary Information.

## Data Availability

The datasets used and/or analysed during the current study available from the corresponding author on reasonable request.
