# Supplementary material for: Probing the design principles of photosynthetic systems through fluorescence noise measurement
Source: Sci Rep. 2024 Jun 16;14:13877. doi: 10.1038/s41598-024-64068-7 (PMC11637105; doi:10.1038/s41598-024-64068-7)
Supplement: Supplementary file 1 — Supplementary Information. [file 41598_2024_64068_MOESM1_ESM.docx]

Probing the design principles of photosynthetic systems through fluorescence noise measurement

Naama Maroudas-Sklare# ^a,b^, Naama Goren# ^a^, Shira Yochelis ^a^, Grzegorz Jung ^c,d^, Nir Keren ^b^, Yossi Paltiel* ^a^

#Equal contribution

^a^ Department of Applied Physics, Hebrew University of Jerusalem, Jerusalem, Israel

^b^ Department of Plant & Environmental Sciences, The Alexander Silberman Institute of Life Sciences, Hebrew University of Jerusalem, Jerusalem, Israel

^c^ Department of Physics, Ben Gurion University of the Negev, 84105 Beer Sheva, Israel

^d^ Instytut Fizyki PAN, 02668 Warszawa, Poland

* Corresponding author: Yossi Paltiel, Department of Applied Physics, Hebrew University of Jerusalem, Jerusalem 91904, Israel

Email: paltiel@mail.huji.ac.il


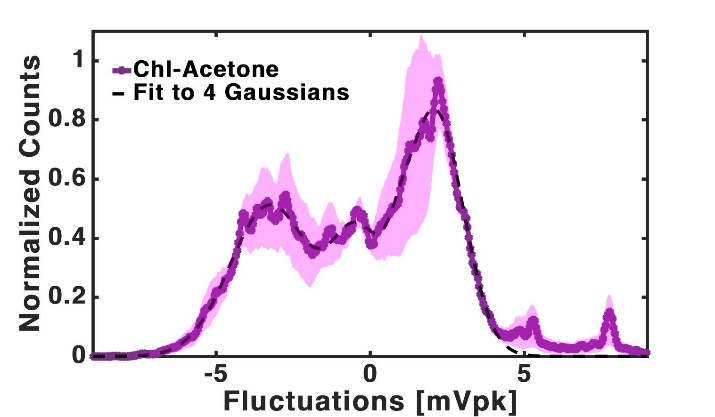

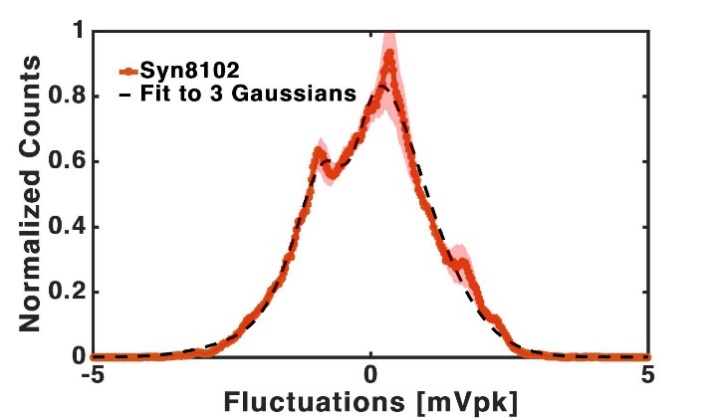

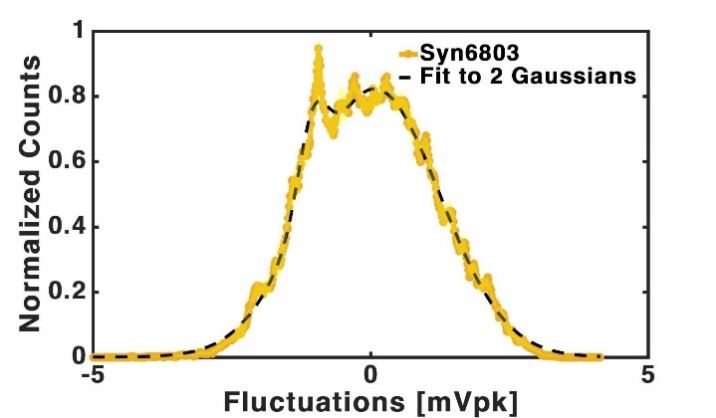

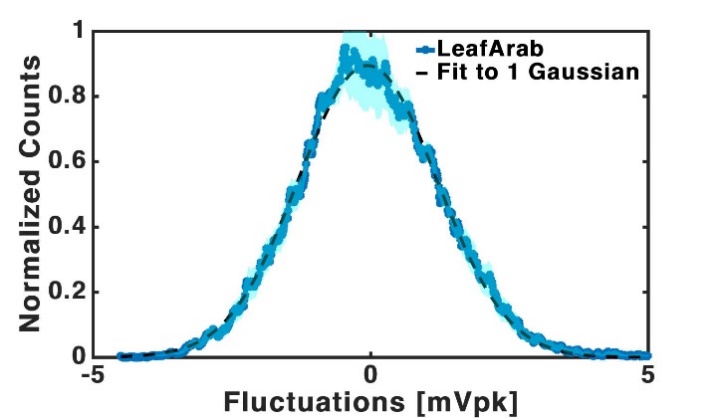

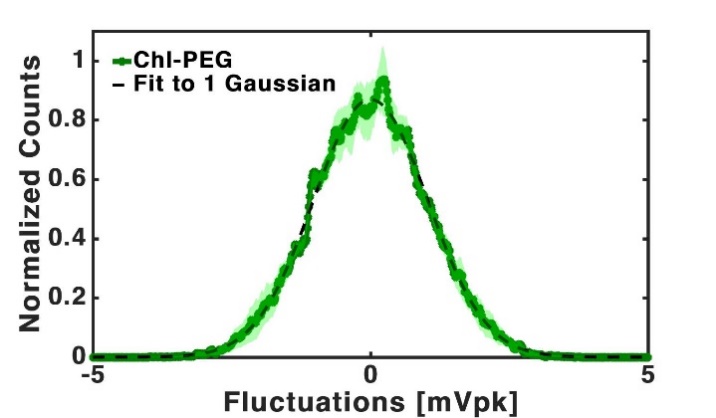

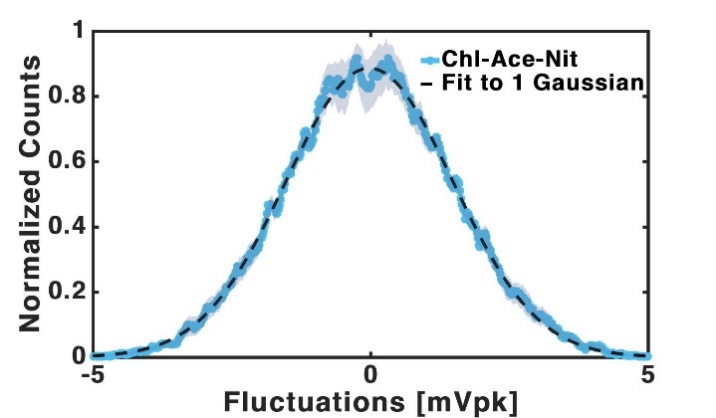


A

B

C

D

E

F


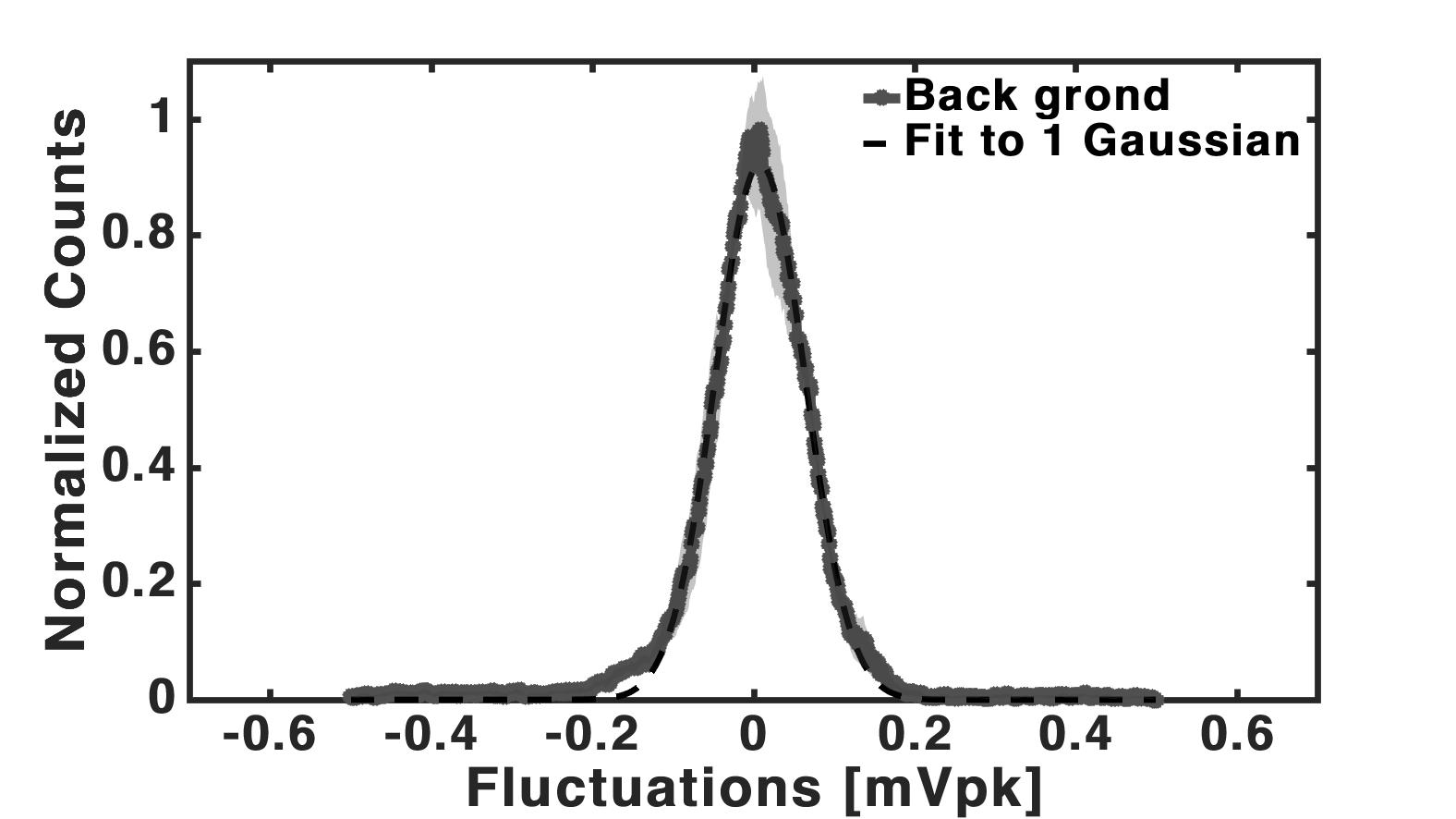


G

**Figure S1: Histograms for all samples:** (A) Chlorophyll in a PEG solution (Chl-PEG), (B) Chlorophyll in an Acetone solution after Nitrogen bubbling (Chl-Ace-Nit), (C) Leaves from *Arabidopsis thaliana* plants (LeafArb), (D) Syn6803, (E) Syn 8102, (F) Chlorophyll in an Acetone solution (Chl-Acetone) and (G) Background of the setup without any sample. Each histogram is an average of four measurement sets, shade area notes the averaging error. Each histogram was fitted to a gaussian model with different degrees shown in dashed black line. All coefficient of gaussian fits shown in table S2 below.

|  | Degree | a | b | c | Set |
| --- | --- | --- | --- | --- | --- |
| Chl-PEG | **1** | 8.69E-01 | 1.52E-06 | 1.46E-03 | 1 |
| Chl-Ace-Nit | **1** | 8.86E-01 | -2.00E-05 | 2.13E-03 | 1 |
| LeafArb | **1** | 8.94E-01 | -5.19E-05 | 1.77E-03 | 1 |
| Syn6803 | **2** | 2.37E-01 | -1.05E-03 | 3.62E-04 | 1 |
|  |  | 8.23E-01 | 7.35E-05 | 1.66E-03 | 2 |
| Syn8102 | **3** | 9.88E-01 | -2.95E-04 | 1.60E-03 | 1 |
|  |  | -2.81E-01 | -3.87E-04 | 4.26E-04 | 2 |
|  |  | -2.57E-01 | -1.42E-03 | 1.06E-03 | 3 |
| Chl-Acetone | **4** | 8.33E-01 | 2.09E-03 | 1.31E-03 | 1 |
|  |  | 8.61E-02 | 9.41E-04 | 5.33E-04 | 2 |
|  |  | 5.11E-01 | -3.24E-03 | 1.84E-03 | 3 |
|  |  | 3.83E-01 | -4.33E-04 | 1.15E-03 | 4 |
| Back ground | 1 | 9.22E-01 | 8.40E-06 | 7.82E-05 | 1 |

**Table 1: Fit coefficients for all histograms:** All histograms were fitted to gaussian model with different degrees of the form: $\sum_{i=1}^{n} a_{i}e^{[-\left( \frac{x-b_{i}}{c_{i}} \right)^{2}]}$, as shown in figure S1 above.


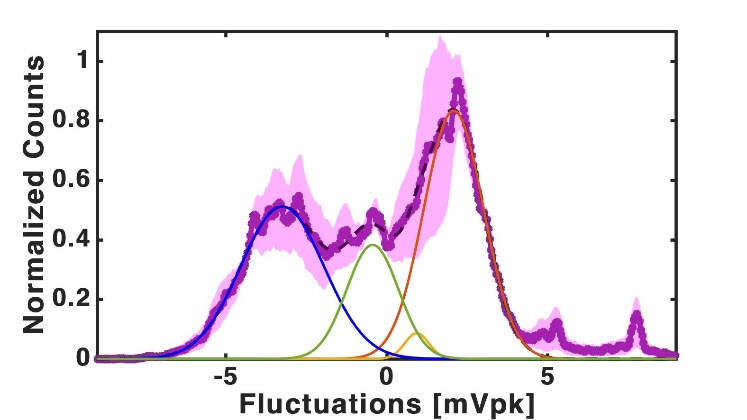


**Figure S2: Gaussian Fit**: Fit components of Chlorophyll in an Acetone solution (Chl-Acetone) histogram as presents in figure S1(F). Histograms coefficients are detailed in table S2.


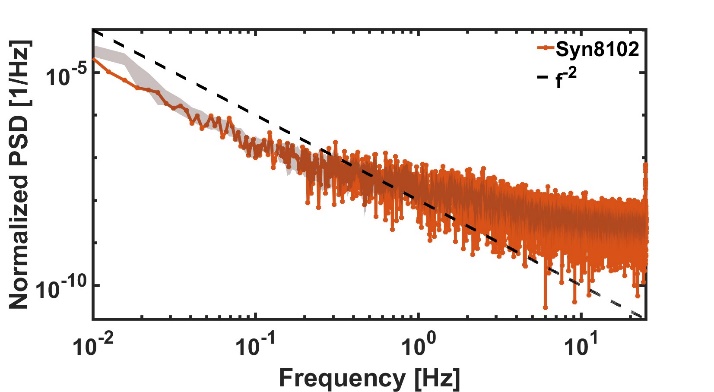

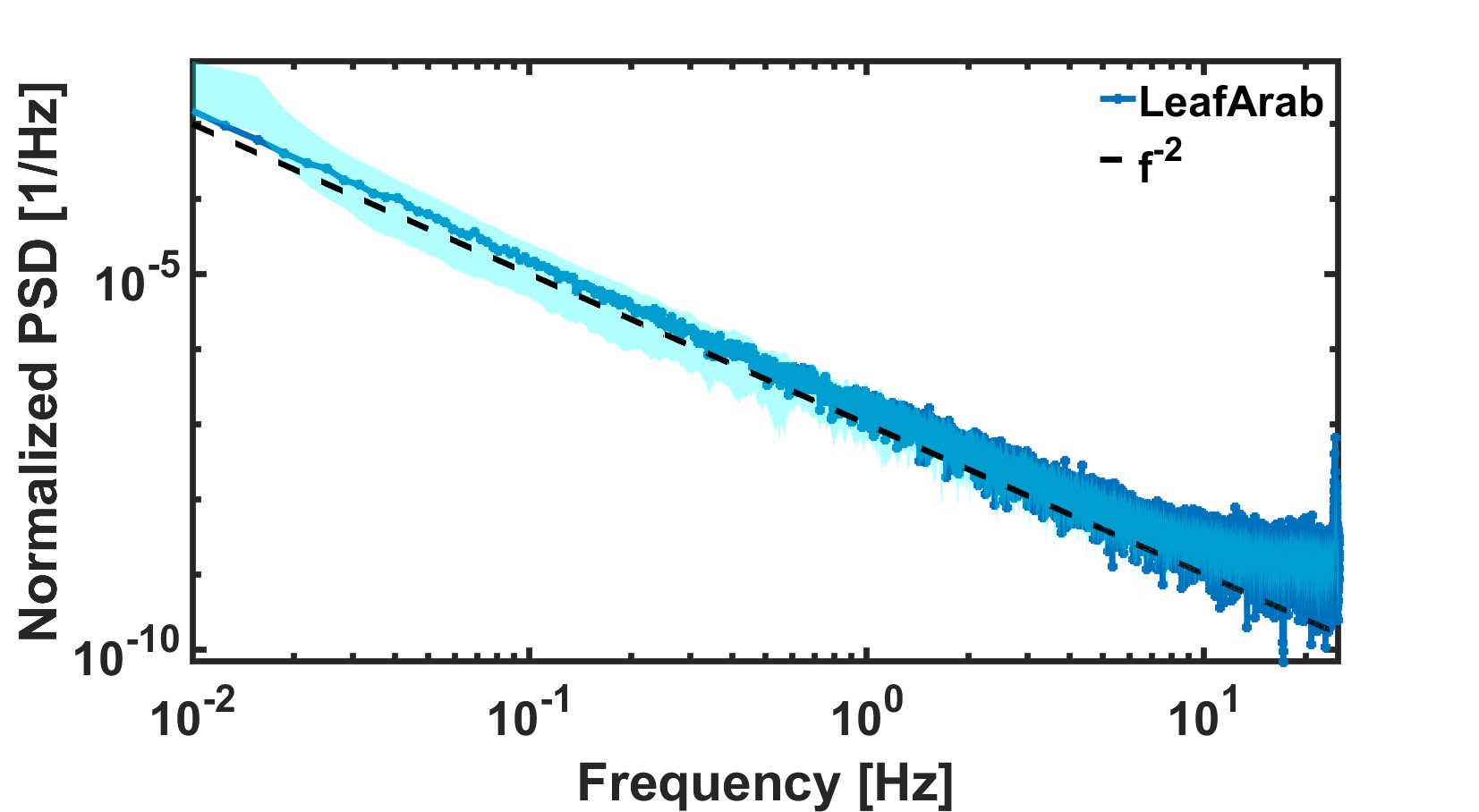

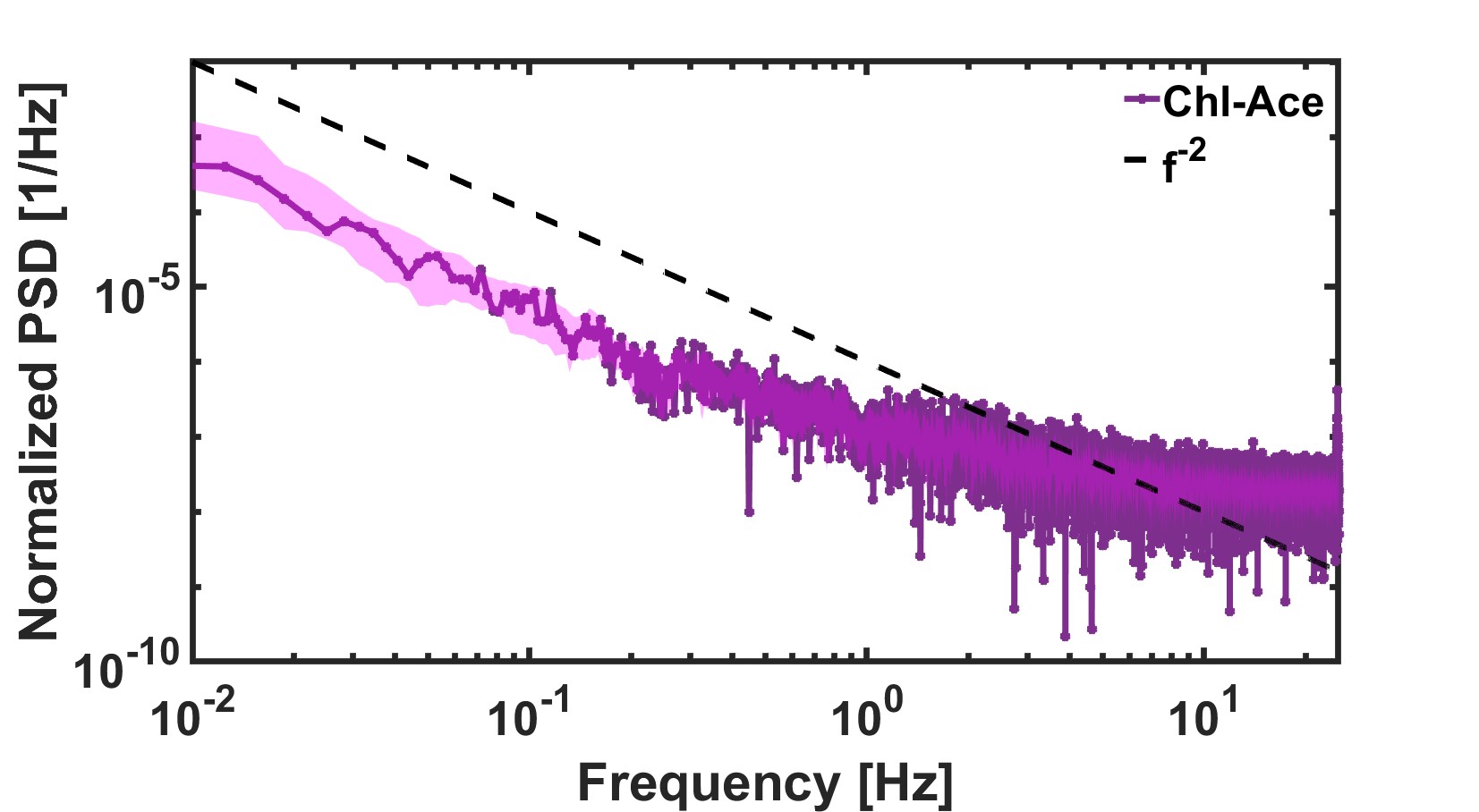

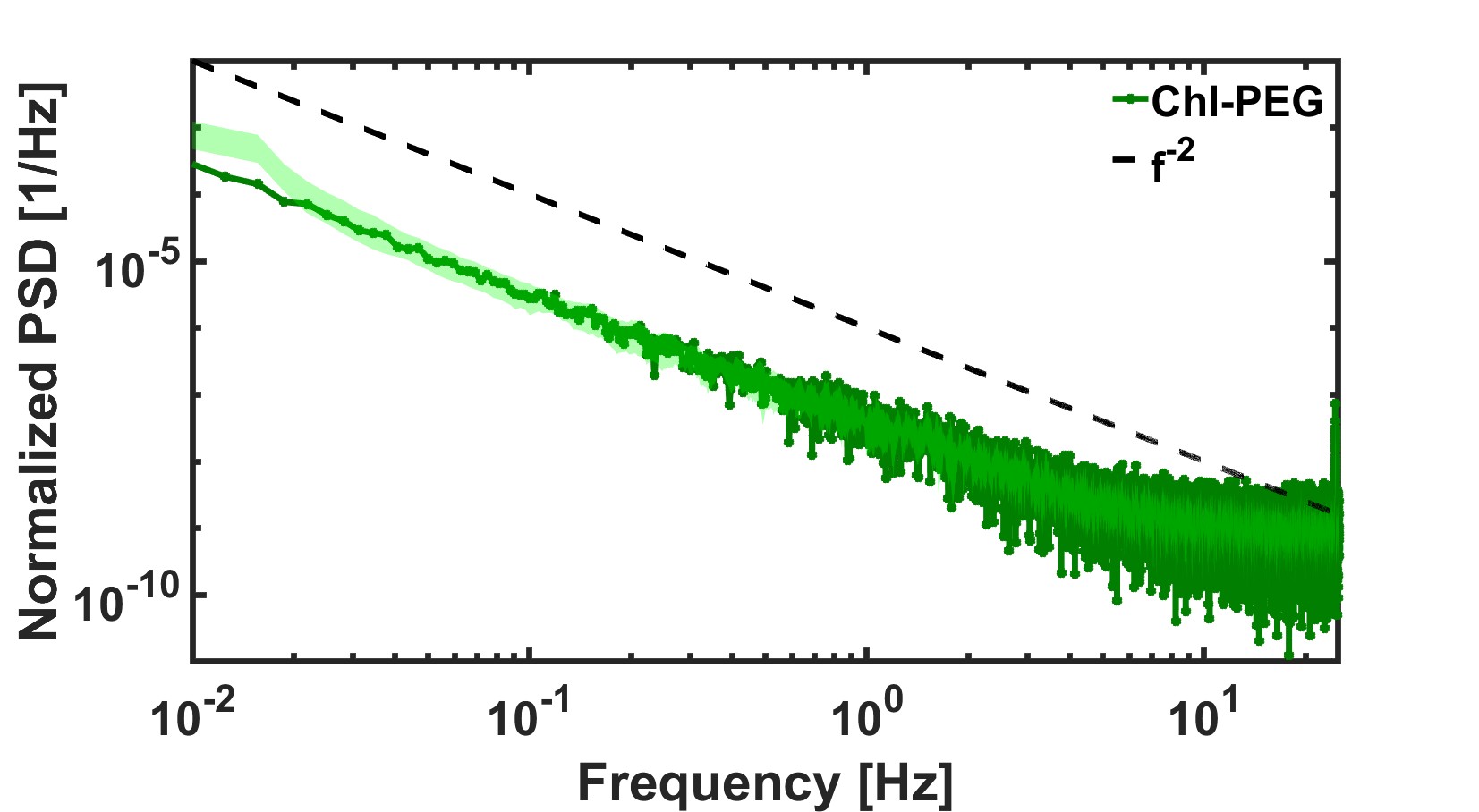

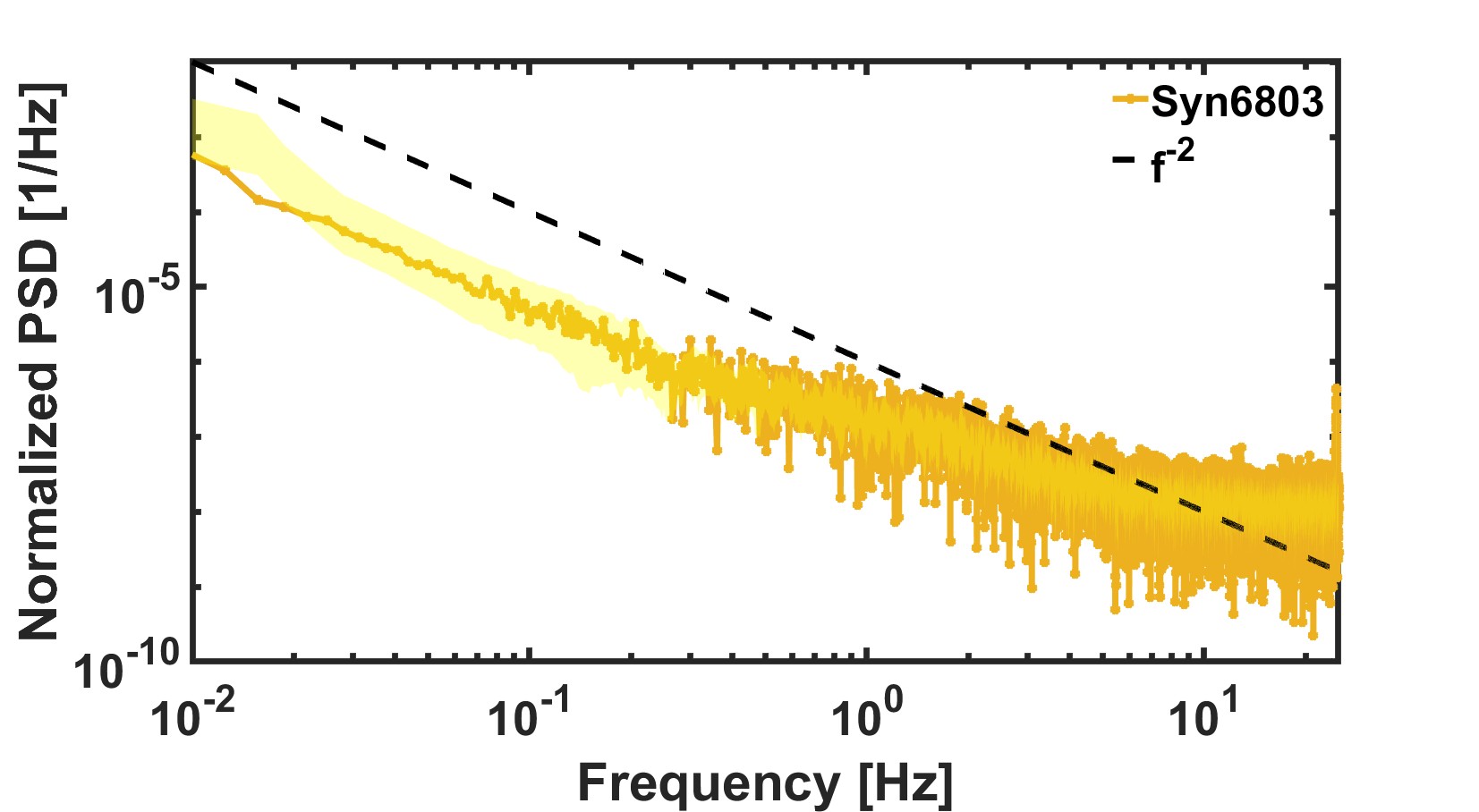

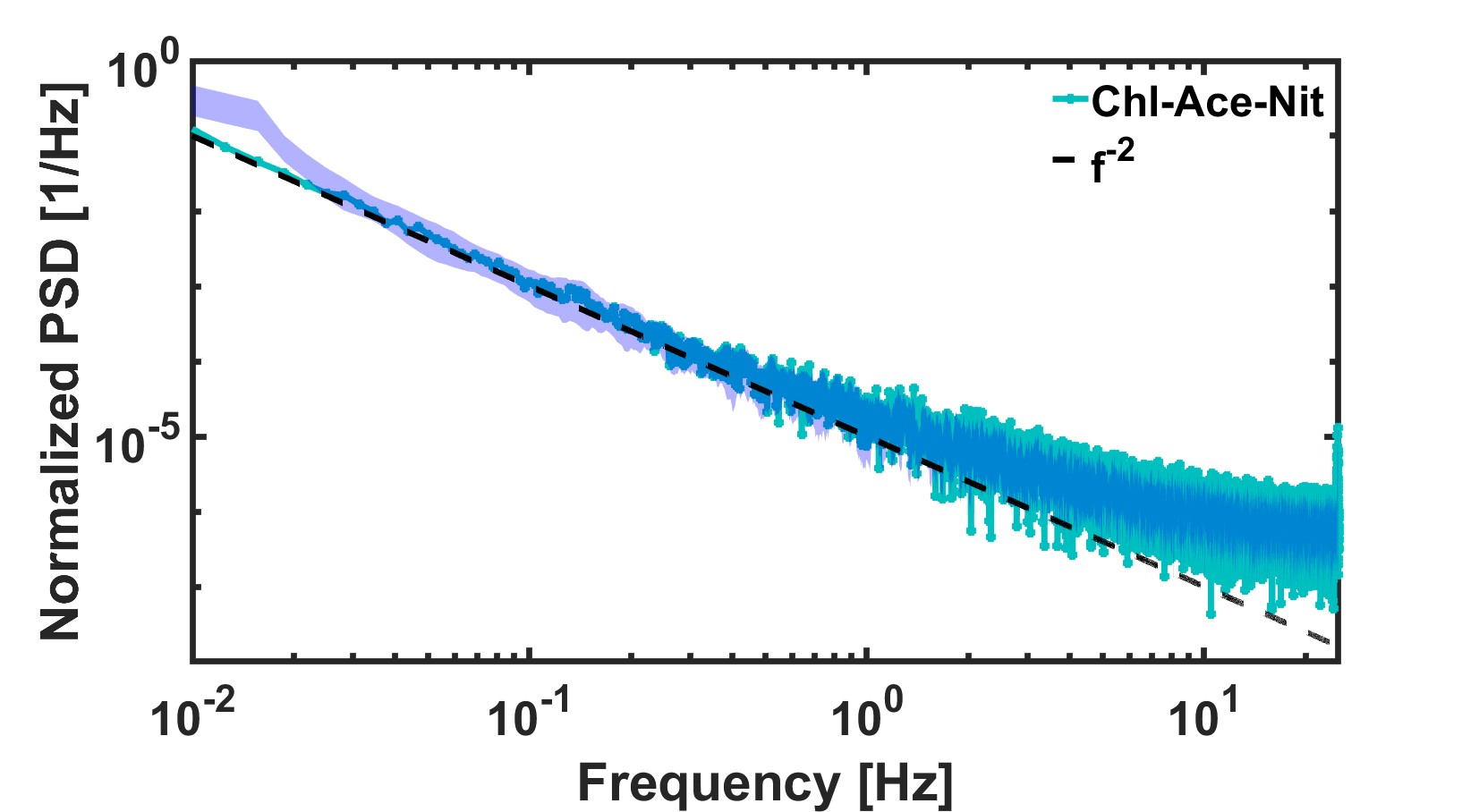


A

B

C

D

E

F


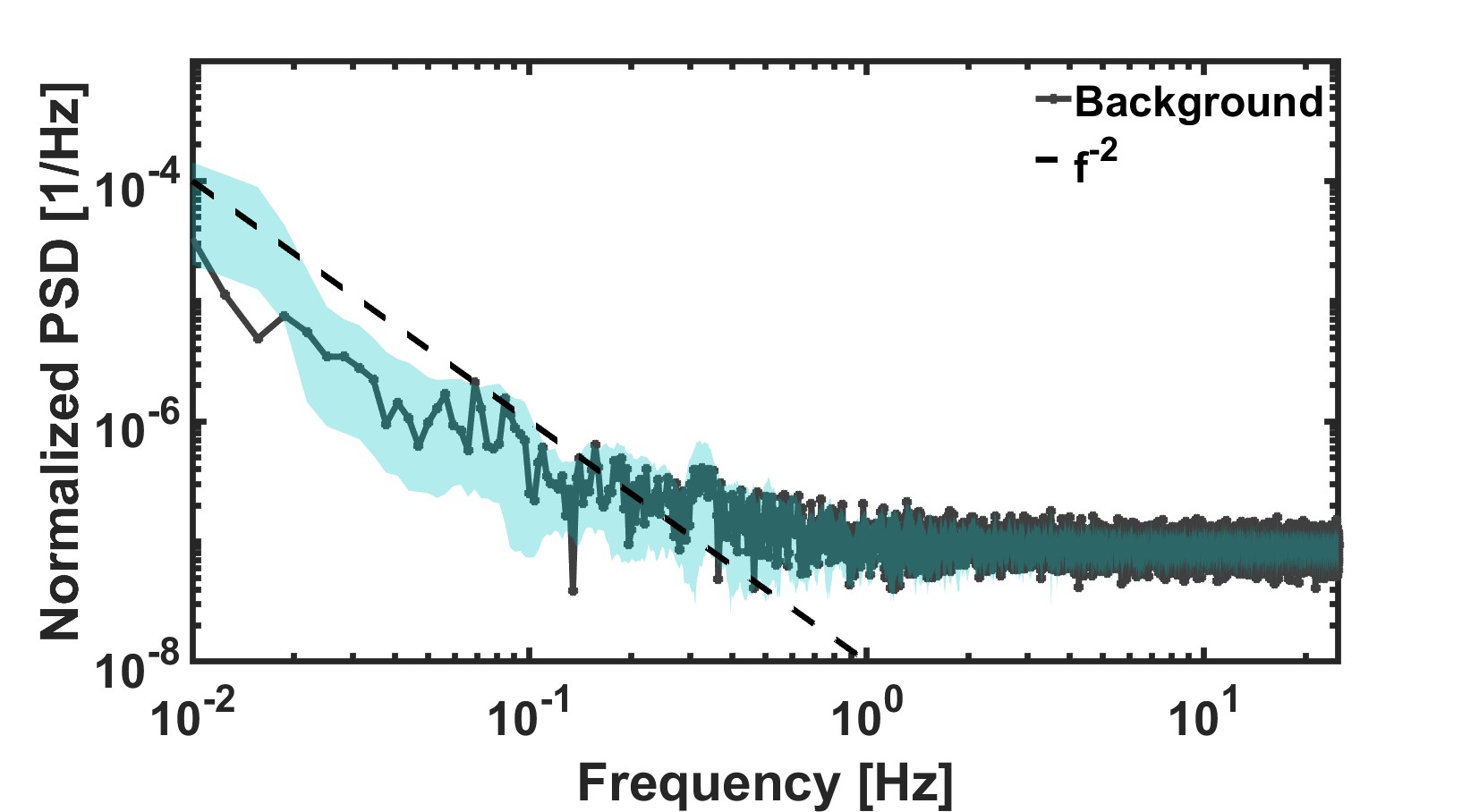


G

**Figure S3: Power spectral density** **of all samples:** (A) Chlorophyll in a PEG solution (Chl-PEG), (B) Chlorophyll in an Acetone solution after Nitrogen bubbling (Chl-Ace-Nit), (C) Leaves from *Arabidopsis thaliana* plants (LeafArb), (D) Syn6803, (E) Syn 8102, (F) Chlorophyll in an Acetone solution (Chl-Acetone) and (G) Background of the setup without any sample. Dashed black line present a first order power law fit $f^{-2}$. Shade area notes the averaging error.

**
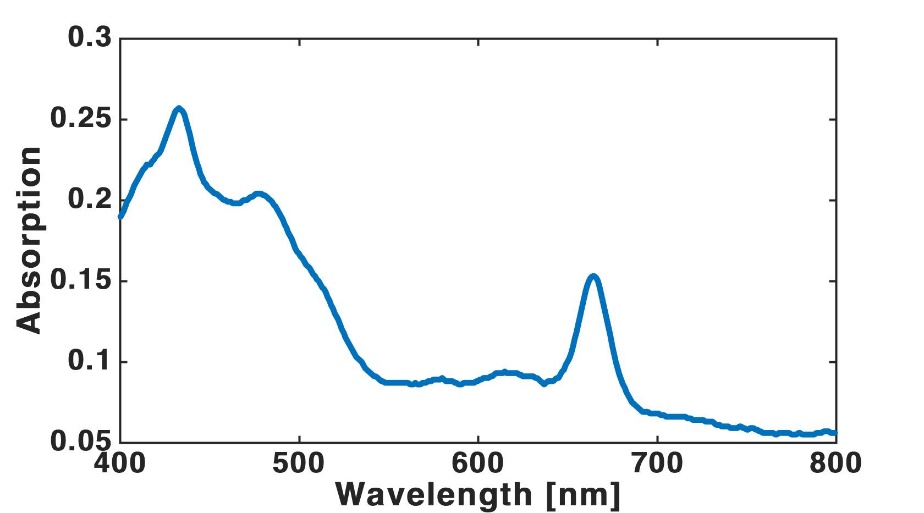
**

**Figure S4: Absorption Signal For Chlorophyll in an Acetone solution (Chl-Acetone):** According to Pora et al ^1^ the peak of chlorophyll a in 80% acetone is at 663.6 nm. Jeffery 1975 ^2^ has 664.3 nm. Our adsorption measurement presents a peak at 664 nm. In a protein context, chlorophylls are hydrogen bonded to the protein framework. Bond energy is the case of the red shift of the chlorophyll a peak.

References

1. Porra, R. J., Thompson, W. A. & Kriedemann, P. E. Determination of accurate extinction coefficients and simultaneous equations for assaying chlorophylls a and b extracted with four different solvents: verification of the concentration of chlorophyll standards by atomic absorption spectroscopy. *Biochim. Biophys. Acta BBA - Bioenerg.* **975**, 384–394 (1989).

2. Jeffrey, S. W. & Humphrey, G. F. New spectrophotometric equations for determining chlorophylls a, b, c1 and c2 in higher plants, algae and natural phytoplankton. *Biochem. Physiol. Pflanz.* **167**, 191–194 (1975).
